# Early Emergence of Ethnic Differences in Type 2 Diabetes Precursors in the UK: The Child Heart and Health Study in England (CHASE Study)

**DOI:** 10.1371/journal.pmed.1000263

**Published:** 2010-04-20

**Authors:** Peter H. Whincup, Claire M. Nightingale, Christopher G. Owen, Alicja R. Rudnicka, Ian Gibb, Catherine M. McKay, Angela S. Donin, Naveed Sattar, K. George M. M. Alberti, Derek G. Cook

**Affiliations:** 1 Division of Community Health Sciences, St George's, University of London, London, United Kingdom; 2 Department of Clinical Biochemistry, Newcastle Hospitals NHS Trust, Newcastle, United Kingdom; 3 BHF Glasgow Cardiovascular Research Centre, University of Glasgow, Glasgow, United Kingdom; 4 Division of Medicine, Imperial College, London, United Kingdom; Lund University Hospital, Sweden

## Abstract

**Background:**

Adults of South Asian origin living in the United Kingdom have high risks of type 2 diabetes and central obesity; raised circulating insulin, triglyceride, and C-reactive protein concentrations; and low HDL-cholesterol when compared with white Europeans. Adults of African-Caribbean origin living in the UK have smaller increases in type 2 diabetes risk, raised circulating insulin and HDL-cholesterol, and low triglyceride and C-reactive protein concentrations. We examined whether corresponding ethnic differences were apparent in childhood.

**Methods and Findings:**

We performed a cross-sectional survey of 4,796 children aged 9–10 y in three UK cities who had anthropometric measurements (68% response) and provided blood samples (58% response); ethnicity was based on parental definition. In age-adjusted comparisons with white Europeans (*n* = 1,153), South Asian children (*n* = 1,306) had higher glycated haemoglobin (HbA1c) (% difference: 2.1, 95% CI 1.6 to 2.7), fasting insulin (% difference 30.0, 95% CI 23.4 to 36.9), triglyceride (% difference 12.9, 95% CI 9.4 to 16.5), and C-reactive protein (% difference 43.3, 95% CI 28.6 to 59.7), and lower HDL-cholesterol (% difference −2.9, 95% CI −4.5 to −1.3). Higher adiposity levels among South Asians (based on skinfolds and bioimpedance) did not account for these patterns. Black African-Caribbean children (*n* = 1,215) had higher levels of HbA1c, insulin, and C-reactive protein than white Europeans, though the ethnic differences were not as marked as in South Asians. Black African-Caribbean children had higher HDL-cholesterol and lower triglyceride levels than white Europeans; adiposity markers were not increased.

**Conclusions:**

Ethnic differences in type 2 diabetes precursors, mostly following adult patterns, are apparent in UK children in the first decade. Some key determinants operate before adult life and may provide scope for early prevention.

*Please see later in the article for the Editors' Summary*

## Abstract 2

Peter Whincup and colleagues carry out a cross-sectional study examining ethnic differences in precursors of of type 2 diabetes among children aged 9–10 living in three UK cities.

## Editors' Summary

**Background:**

Worldwide, nearly 250 million people have diabetes, and the number of people affected by this chronic disease is increasing rapidly. Diabetes is characterized by dangerous amounts of sugar (glucose) in the blood. Blood sugar levels are normally controlled by insulin, a hormone that the pancreas releases when blood sugar levels rise after eating (digestion of food produces glucose). In people with type 2 diabetes (the most common type of diabetes), blood sugar control fails because the fat and muscle cells that usually respond to insulin by removing sugar from the blood become less responsive to insulin (insulin resistant). Type 2 diabetes can be controlled with diet and exercise, and with drugs that help the pancreas make more insulin or that make cells more sensitive to insulin. Long-term complications of diabetes include kidney failure, blindness, nerve damage, and an increased risk of developing cardiovascular problems, including heart disease and stroke.

**Why Was This Study Done?:**

South Asians and African-Caribbeans living in Western countries tend to have higher rates of type 2 diabetes than host populations. South Asian adults living in the UK, for example, have a 3-fold higher risk of developing type 2 diabetes than white Europeans. They also have higher fasting blood levels of glucose, insulin and triglycerides (a type of fat), higher blood levels of “glycated hemoglobin” (HbA1c; an indicator of average of blood-sugar levels over time), more body fat (increased adiposity), raised levels of a molecule called C-reactive protein, and lower levels of HDL-cholesterol (another type of fat) than white Europeans. Most of these “diabetes precursors” (risk factors) are also seen in black African-Caribbean adults living in the UK except that individuals in this ethnic group often have raised HDL-cholesterol levels and low triglyceride levels. Ethnic differences in type 2 diabetes precursors are also present in adolescents, but the extent to which they are present in childhood remains unclear. Knowing this information could have implications for diabetes prevention. In this population-based study, therefore, the researchers investigate patterns of diabetes precursors in 9- to 10-year-old UK children of white European, South Asian, and black African-Caribbean origin.

**What Did the Researchers Do and Find?:**

The researchers enrolled nearly 5,000 children (including 1,153 white European, 1,306 South Asian and 1,215 black African-Caribbean children) from primary schools with high prevalences of ethnic minority pupils in London, Birmingham, and Leicester in the Child Heart and Health study in England (CHASE). They measured and weighed more than two-thirds of the enrolled children and determined their adiposity. They also took blood samples for measurement of diabetes precursors from nearly two-thirds of the children. The recorded ethnicity of each child was based on parental definition. The researchers' analysis of these data showed that, compared with white Europeans, South Asian children had higher levels of HbA1c, insulin, triglycerides, and C-reactive protein but lower HDL-cholesterol levels. In addition, they had higher adiposity levels than the white European children, but this did not account for the observed differences in the other diabetes precursors. Black African-Caribbean children also had higher levels of HbA1c, insulin, and C-reactive protein than white European children, although the differences were smaller than those between South Asians and white Europeans. Similar to black African-Caribbean adults, however, children of this ethnic origin had higher HDL-cholesterol and lower triglyceride levels than white Europeans.

**What Do These Findings Mean?:**

These findings indicate that ethnic differences in diabetes precursors are already present in apparently healthy children before they are 10 years old. Furthermore, most of the ethnic differences in diabetes precursors seen among the children follow the pattern seen in adults. Although these findings need confirming in more children, they suggest that the ethnic differences in type 2 diabetes susceptibility first described in immigrants to the UK are persisting in UK-born South Asian and black African-Caribbean children. Most importantly, these findings suggest that some of the factors thought to be responsible for ethnic differences in type 2 diabetes—for example, varying levels of physical activity and dietary differences—are operating well before adult life. Interventions that target these factors early could, therefore, offer good opportunities for diabetes prevention in high-risk ethnic groups, provided such interventions are carefully tailored to the needs of these groups.

**Additional Information:**

Please access these Web sites via the online version of this summary at http://dx.doi.org/10.1371/journal.pmed.1000263.

- The International Diabetes Federation provides information about all aspects of diabetes (in English, French and Spanish)
- The US National Diabetes Information Clearinghouse provides detailed information about diabetes for patients, health-care professionals and the general public, including information on diabetes in specific US populations (in English and Spanish)
- The UK National Health Service also provides information for patients and carers about type 2 diabetes (in several languages)
- MedlinePlus provides links to further resources and advice about diabetes (in English and Spanish)
- The US Agency for Healthcare Research and Quality has a fact sheet on diabetes disparities among racial and ethnic minorities

## Introduction

Asian populations, both in Asia and in diaspora populations, are facing an epidemic of type 2 diabetes [1]. South Asian adults living in the UK have markedly higher (approximately 3-fold) risks of type 2 diabetes than white Europeans [2]–[4]. In population-based studies, they generally have raised plasma glucose and glycated haemoglobin (HbA1c), fasting insulin, and triglyceride concentrations, higher levels of adiposity (particularly central adiposity), but lower HDL-cholesterol concentrations [2]–[5]. C-reactive protein levels (strongly associated with insulin resistance and type 2 diabetes risk [6],[7]) are also elevated [6],[8]. Among UK South Asians, diabetes prevalence tends to be higher among Bangladeshis and Pakistanis than Indians [3],[5],[9].

Black African-Caribbean adults living in the UK are also at increased risk (approximately 2-fold) of type 2 diabetes when compared with white Europeans [3],[4],[10]. In population-based studies, black African-Caribbeans have generally had raised plasma glucose, HbA1c, and fasting insulin concentrations, though less so than South Asians [3],[4],[10]. Paradoxically, black African-Caribbeans have higher mean HDL-cholesterol levels and lower levels of triglyceride and C-reactive protein than white Europeans [3],[4],[10],[11]. Adiposity (particularly general adiposity) tends to be increased [3],[10]. These disease and risk factor patterns have generally been similar in black adults of both African and Caribbean origins [3],[4].

Early life exposures have been implicated in the aetiology of type 2 diabetes [12]. It is now recognized that the higher risks of type 2 diabetes among Asians (including South Asians) are apparent in early adult life and adolescence both in the US [13] and in the UK [14]. It has been suggested that population-wide differences in diabetes precursors between South Asians and white Europeans could be apparent in childhood [15],[16]. We report here the results of a large-scale population-based study investigating patterns of adiposity, glucose, insulin, related blood lipids, and C-reactive protein among 9- to 10-y-old UK children of white European and South Asian origin, and specifically including children of Bangladeshi, Pakistani, and Indian origins. The study also aimed to investigate risk factor patterns among black African-Caribbean children (including both African and Caribbean origins).

## Methods

The Child Heart and Health Study in England (CHASE Study) is a school-based investigation of the health of British children aged 9–10 y old, living in London, Leicester, and Birmingham. Ethical approval was obtained from the Multicentre Research Ethics Committee (Wales); the study was carried out in accordance with the principles expressed in the Declaration of Helsinki. School-level information on pupil ethnicity was provided by the UK Government Department for Education and Skills. The school sampling frame included all state primary schools in London, Birmingham, and Leicester with between 15% and 50% pupils of white European origin. Two separate random samples, each of 100 schools, were taken. The first included schools in which pupils of South Asian origin comprised 20%–80% of all pupils, stratified by Indian, Pakistani, and Bangladeshi origin. The second included schools in which pupils of black African-Caribbean origin comprised 20%–80% of all pupils, stratified by African and Caribbean origin. All Head Teachers were approached by the Principal Investigator and invited to participate; 140 (70%) agreed. Non-participating schools were replaced by a school from the sampling frame with a similar ethnic mix and in the same or a neighbouring borough. The combined sample included 183 London (between 2 and 19 schools in each of 20 London boroughs), 14 Birmingham, and 3 Leicester schools. Depending on school size, either one or two classes of children were invited to participate.

The Study Research Co-ordinator visited schools in advance to explain details of the study to pupils (and parents where possible) and to answer their questions. Informed written consent was obtained from pupils' parent or guardian. A single survey team including three trained research nurses and a support fieldworker carried out all survey measurements during school terms between October 2004 and February 2007; two weekly visits were made to schools in North-West, North-East, or South London in rotation, with periodic visits to Birmingham and Leicester schools. Participating children were asked to fast overnight and provided a blood sample between 8.30 and 10.30 a.m.; breakfast was then provided. Height was measured to the last complete millimetre with a portable stadiometer (Chasmors Ltd, London, UK) and weight with an electronic digital scale (Tanita Inc, Tokyo, Japan); ponderal index (weight kg/height m^3^) was used because it was substantially independent of height in this study population. Waist circumference was measured at the midpoint between the lower margin of the ribs and the pelvic crest in the mid-axillary line. Right-sided skinfolds (biceps, triceps, subscapular, suprailiac) were measured; analyses are based on the sum of the four measurements. Leg to arm bioimpedance was measured using the Bodystat 1500 bioimpedance monitor (Bodystat Ltd, Isle of Man, UK); fat mass was derived using equations derived specifically for children using dual energy X-ray absorptiometry (DXA) validation [17] and presented as a fat mass index (fat mass/height^5^), which was independent of height. Seated blood pressure was measured twice in the right arm after 5 min of rest using an Omron 907 blood pressure recorder, with an appropriately sized cuff. A simplified assessment of pubertal status was made using Tanner scales [18]. Participating children provided questionnaire information on parental and grandparental country of birth and reported any current health problems. The parent or guardian was asked to provide information on the ethnicity of both parents and that of the child, coded using a classification similar to the 2001 UK Census, and on their occupation, coded using the National Statistics Standard Occupational Classification (SOC-2000) [19]. Ethnicity of the children was defined using the ethnicity of both parents or (if not available) the ethnicity of the child; in a small proportion of cases in which parental information was not available (1%), child information on the place of birth of parents and grandparents was used to define ethnic origin. In the analyses presented, “white European” includes children whose ethnic origin was defined as “white British,” “white Irish,” and “white European” (or a combination of these). “South Asian” includes “Indian,” “Pakistani,” “Bangladeshi,” and “Sri Lankan” (or a combination of these). “Other Asian” includes “Asian other” and “other” with a specified Asian place of origin (mainly Afghanistan, China, and Turkey). “Black African-Caribbean” includes “black African,” “black Caribbean,” “black British,” and “black other” (or a combination of these). The “other” ethnic group includes all other categories of individual and mixed ethnic origins. The ethnic subcategories “Indian,” “Pakistani,” and “Bangladeshi” include children whose parents both originated in the same county; “black African” and “black Caribbean” groups include those who originated in the same region.

All laboratory analyses were carried out blind to participant ethnicity. Analyses of HbA1c, glucose, and blood lipids were carried out in the Department of Clinical Biochemistry, Newcastle Hospitals NHS Trust, which received blood samples within 48 h of collection. Glucose was measured in plasma using the hexokinase method. HbA1c was measured in whole blood by ion exchange high performance liquid chromatography; HbA1c values were recalculated to adjust for abnormal haemoglobin variants or for increased amounts of normal variant fetal haemoglobin (HbF) where present. Triglyceride and HDL-cholesterol were measured in serum using an Olympus auto-analyser. Serum, separated and frozen on dry ice after collection, were used for measurement of insulin (Department of Medicine, University of Newcastle, UK) using an ELISA method which does not cross-react with proinsulin [20] and C-reactive protein, which was assayed by ultra-sensitive nephelometry (Dade Behring, Milton Keynes, UK). The homeostasis model assessment (HOMA) model equations were used to provide an estimate of insulin resistance [21].

### Statistical Methods

Statistical analyses were carried out using STATA/SE software (Stata/SE 10 for Windows, StataCorp LP, College Station, TX, USA). All main outcome variables followed approximately log-normal distributions, and logarithmic transformation was undertaken; ethnic differences in these variables were expressed as percentages. Multilevel linear regression models fitting school as a random effect were used to take account of the natural clustering of children within school and to provide adjusted means and adjusted ethnic differences in risk markers. Tests of interaction were carried out to examine whether ethnic differences were modified by gender. Heterogeneity between ethnic subcategories within each main ethnic group was tested using likelihood ratio tests. All analyses were adjusted for age in quartiles, observer (physical measurements only), gender, and month; adiposity measures, where included for adjustment, were fitted as continuous variables.

## Results

**Figure 1:**
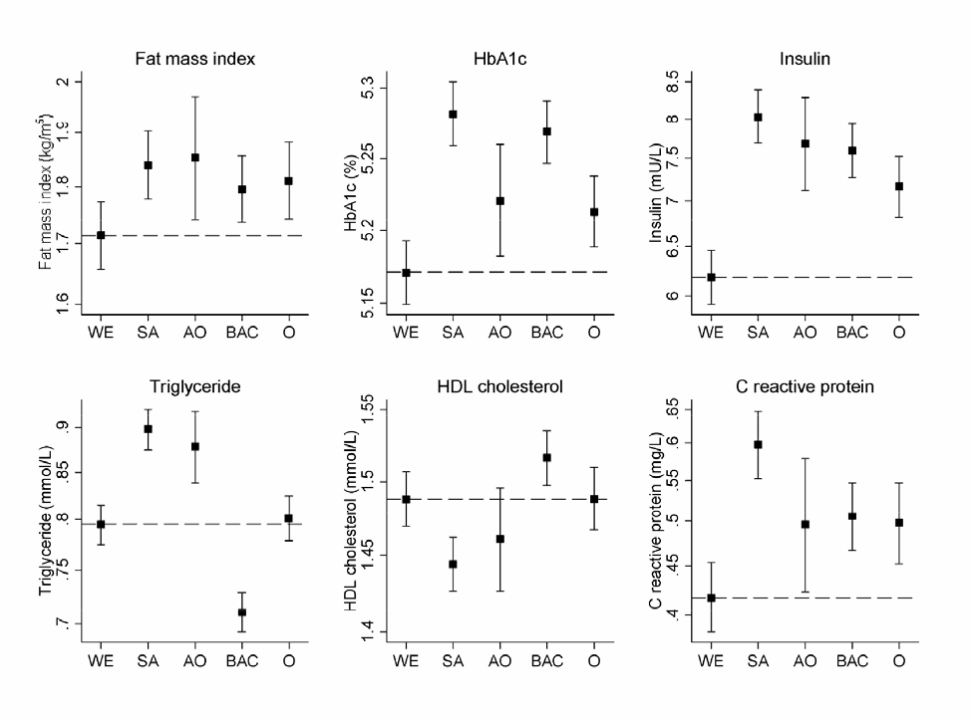
Geometric means and 95% confidence intervals for fat mass index and selected blood markers by ethnic group. WE, white European; SA, South Asian; OA, other Asian; BAC, black African-Caribbean; O, other. Numbers of subjects in each figure correspond to those in Table 2.

Among 8,641 pupils invited, 5,887 (68%) took part and 5,004 (85% of participants) provided blood samples; 7 children with type 1 diabetes were excluded and analyses are based on 4,796 participants (2,325 boys and 2,471 girls) who fasted overnight. The mean age of participants was 10.0 (SD 0.4) y; this did not differ between ethnic groups (*p* = 0.84). The study included similar numbers of children of white European, South Asian, and black African-Caribbean origin (*n* = 1,153, 1,306, and 1,215, respectively), with a smaller number of other Asian children (*n* = 294) and of other ethnic origins (*n* = 828). Participation rates were broadly similar among white Europeans, South Asians, other Asians, and other ethnic groups (69%, 72%, 74%, and 68%, respectively) and slightly lower among black African-Caribbeans (66%). Proportions of children providing blood samples were also higher among white Europeans, South Asians, other Asians, and other ethnic groups (88%, 87%, 86%, and 88%, respectively) and lower among black African-Caribbeans (79%). Physical measurement and blood analyte mean values are shown in Table 1 for boys and girls. Girls were slightly taller and heavier on average and had higher mean ponderal index, sum of skinfolds, fat mass index, waist circumference, fasting insulin, insulin resistance, and triglyceride levels; boys had higher levels of HDL-cholesterol and blood glucose. The mean risk factor values in the main ethnic groups are shown in Table 2 and for selected risk factors in Figure 1; differences between Asian groups and white Europeans are shown in Table 3, and differences between black African-Caribbeans and white Europeans are shown in Table 4.

**Table 1:** Physical measurements and blood markers: By gender.

Measurement/Marker	Boys (*n* = 2,325)		Girls (*n* = 2,471)		*p* Value (Difference)
	Geometric Mean	IQR	Geometric Mean	IQR	
Height (cm)	139.7	9.2	140.3	10.1	0.001
Weight (kg)	35.5	11.2	36.6	12.6	<0.0001
Ponderal Index (kg/m^3^)	13.0	2.8	13.2	3.2	0.002
Sum of skinfolds (mm)	36.6	30.0	45.5	35.4	<0.0001
Fat mass index (kg/m^5^)	1.69	1.15	1.89	1.28	<0.0001
Waist circumference (cm)	63.3	11.9	64.3	13.5	<0.0001
HbA1c (%)	5.2	0.4	5.2	0.4	0.17
Glucose (mmol/L)	4.6	0.5	4.5	0.4	<0.0001
Insulin (mU/L)	6.4	5.1	8.1	6.2	<0.0001
Insulin resistance	0.81	0.70	1.02	0.80	<0.0001
Triglyceride (mmol/L)	0.76	0.40	0.85	0.40	<0.0001
HDL cholesterol (mmol/L)	1.53	0.50	1.44	0.40	<0.0001
C-reactive protein (mg/L)	0.44	0.79	0.57	1.17	<0.0001

Geometric means adjusted for age quartiles, observer (physical measurements), month, and a random effect for school. Interquartile ranges (IQR) are based on raw data. Missing values: Fat mass index (*n* = 62), insulin and insulin resistance (*n* = 121), and C-reactive protein (*n* = 188).

**Table 2:** Physical measurements and blood markers: By ethnic group.

Measurement/Marker	White European (*n* = 1,153)	South Asian (*n* = 1,306)	Other Asian (*n* = 294)	Black African-Caribbean (*n* = 1,215)	Other (n = 828)
	Geometric Mean (95% CI)	Geometric Mean (95% CI)	Geometric Mean (95% CI)	Geometric Mean (95% CI)	Geometric Mean (95% CI)
Height (cm)	138.9 (138.5, 139.3)	138.5 (138.1, 138.9)	138.3 (137.5, 139.1)	143.0 (142.6, 143.4)	140.0 (139.5, 140.4)
Weight (kg)	35.2 (34.7, 35.7)	34.3 (33.8, 34.7)	35.3 (34.3, 36.2)	38.8 (38.3, 39.3)	36.5 (35.9, 37.1)
Ponderal Index (kg/m^3^)	13.1 (13.0, 13.3)	12.9 (12.8, 13.0)	13.3 (13.1, 13.6)	13.2 (13.1, 13.4)	13.3 (13.1, 13.4)
Sum of skinfolds (mm)	40.4 (39.2, 41.6)	42.5 (41.3, 43.8)	42.4 (40.0, 44.8)	39.6 (38.4, 40.8)	40.8 (39.5, 42.3)
Fat mass index (kg/m^5^)	1.71 (1.66, 1.77)	1.84 (1.78, 1.90)	1.85 (1.74, 1.97)	1.80 (1.74, 1.86)	1.81 (1.74, 1.88)
Waist circumference (cm)	63.9 (63.4, 64.5)	63.0 (62.5, 63.5)	64.2 (63.2, 65.3)	64.2 (63.7, 64.7)	64.2 (63.6, 64.9)
HbA1c (%)	5.2 (5.1, 5.2)	5.3 (5.3, 5.3)	5.2 (5.2, 5.3)	5.3 (5.2, 5.3)	5.2 (5.2, 5.2)
Glucose (mmol/L)	4.5 (4.5, 4.5)	4.5 (4.5, 4.6)	4.5 (4.5, 4.6)	4.5 (4.5, 4.5)	4.5 (4.5, 4.5)
Insulin (mU/L)	6.2 (5.9, 6.5)	8.0 (7.7, 8.4)	7.7 (7.1, 8.3)	7.6 (7.3, 7.9)	7.2 (6.8, 7.5)
Insulin resistance	0.78 (0.75, 0.81)	1.01 (0.97, 1.06)	0.96 (0.89, 1.04)	0.95 (0.91, 1.00)	0.90 (0.86, 0.95)
Triglyceride (mmol/L)	0.79 (0.77, 0.82)	0.90 (0.87, 0.92)	0.88 (0.84, 0.92)	0.71 (0.69, 0.73)	0.80 (0.78, 0.82)
HDL cholesterol (mmol/L)	1.49 (1.47, 1.51)	1.44 (1.43, 1.46)	1.46 (1.43, 1.50)	1.52 (1.50, 1.53)	1.49 (1.47, 1.51)
C-reactive protein (mg/L)	0.42 (0.38, 0.45)	0.60 (0.55, 0.65)	0.49 (0.42, 0.58)	0.50 (0.47, 0.55)	0.50 (0.45, 0.55)

Geometric means adjusted for age quartiles, gender, observer (physical measurements), month, and a random effect for school. Missing values: Fat mass index (*n* = 62), insulin and insulin resistance (*n* = 121), and C-reactive protein (*n* = 188).

**Table 3:** Ethnic differences in physical measurements and blood markers (South Asian and Asian other minus white Europeans).

Measurement/Marker	All South Asian (*n* = 1,306)		South Asian Subcategories				All Other Asian (*n* = 294)	
			Indian (*n* = 407)	Pakistani (*n* = 473)	Bangladeshi (*n* = 321)			
	% Difference (95% CI)	*p* (Diff)	% Difference (95% CI)	% Difference (95% CI)	% Difference (95% CI)	*p* Value A	% Difference (95% CI)	*p* (Diff)
Height	−0.3 (−0.7, 0.1)	0.16	−0.1 (−0.7, 0.4)	0.1 (−0.4, 0.6)	−1.0 (−1.6, −0.4)	0.004	−0.4 (−1.0, 0.2)	0.18
Weight	−2.5 (−4.4, −0.7)	0.01	−3.4 (−6.0, −0.8)	−1.6 (−4.1, 0.9)	−2.4 (−5.2, 0.6)	0.51	0.3 (−2.6, 3.4)	0.83
Ponderal Index	−1.8 (−3.1, −0.4)	0.01	−3.1 (−5.0, −1.3)	−2.0 (−3.8, −0.2)	0.5 (−1.6, 2.7)	0.02	1.6 (−0.6, 3.8)	0.15
Sum of skinfolds	5.1 (1.1, 9.4)	0.01	3.9 (−1.8, 9.9)	7.1 (1.6, 13.0)	5.2 (−1.2, 12.0)	0.62	4.9 (−1.5, 11.6)	0.14
Fat mass index	7.3 (2.8, 12.0)	0.001	2.6 (−3.5, 9.1)	6.6 (0.6, 13.0)	14.2 (6.7, 22.3)	0.03	8.1 (1.0, 15.6)	0.02
Waist circumference	−1.5 (−2.6, −0.3)	0.01	−2.4 (−4.0, −0.9)	−0.7 (−2.2, 0.8)	−1.3 (−3.0, 0.5)	0.17	0.4 (−1.4, 2.2)	0.67
HbA1c	2.1 (1.6, 2.7)	<0.0001	2.8 (2.0, 3.5)	2.5 (1.8, 3.2)	1.0 (0.2, 1.8)	<0.001	1.0 (0.2, 1.8)	0.02
Glucose	0.8 (0.2, 1.5)	0.01	0.7 (−0.2, 1.6)	1.1 (0.2, 2.0)	1.5 (0.4, 2.5)	0.45	0.8 (−0.2, 1.8)	0.13
Insulin	30.0 (23.4, 36.9)	<0.0001	29.3 (20.1, 39.3)	22.9 (14.6, 31.8)	46.2 (34.6, 58.8)	0.002	24.3 (14.7, 34.7)	<0.0001
Insulin resistance	29.6 (23.1, 36.4)	<0.0001	28.4 (19.4, 38.2)	22.9 (14.7, 31.8)	45.8 (34.3, 58.2)	0.002	23.5 (14.0, 33.8)	<0.0001
Triglyceride	12.9 (9.4, 16.5)	<0.0001	10.8 (6.0, 15.9)	11.7 (7.1, 16.5)	19.5 (13.7, 25.7)	0.02	10.5 (5.2, 16.0)	<0.0001
HDL cholesterol	−2.9 (−4.5, −1.3)	<0.001	−1.5 (−3.8, 0.8)	−1.4 (−3.6, 0.8)	−6.9 (−9.3, −4.4)	<0.001	−1.8 (−4.4, 0.8)	0.17
C-reactive protein	43.3 (28.6, 59.7)	<0.0001	39.0 (19.0, 62.2)	54.3 (33.3, 78.5)	36.1 (14.8, 61.2)	0.32	18.6 (−0.3, 41.1)	0.05

“Ethnic differences” refer to differences from white Europeans and are adjusted for age quartiles, gender observer (physical measurements), month, and a random effect for school. Missing values: Fat mass index (*n* = 62), insulin and insulin resistance (*n* = 121), and C-reactive protein (*n* = 188).

*p* value A = *p* (no difference between South Asian subcategories).

**Table 4:** Ethnic differences in physical measurements and blood markers (black African-Caribbean minus white Europeans).

Measurement/Marker	All Black African-Caribbean (*n* = 1,215)		Black African-Caribbean Subcategories		
			Black African (*n* = 662)	Black Caribbean (*n* = 465)	
	% Difference (95% CI)	*p* (Diff)	% Difference (95% CI)	% Difference (95% CI)	*p* Value B
Height	3.0 (2.6, 3.4)	<0.0001	3.0 (2.5, 3.5)	3.0 (2.5, 3.5)	0.95
Weight	10.3 (8.2, 12.4)	<0.0001	9.1 (6.7, 11.6)	12.0 (9.2, 14.9)	0.07
Ponderal Index	0.9 (−0.4, 2.3)	0.19	−0.1 (−1.7, 1.5)	2.5 (0.7, 4.4)	0.01
Sum of skinfolds	−2.1 (−5.9, 1.9)	0.30	−1.3 (−5.9, 3.4)	−1.1 (−6.2, 4.2)	0.95
Fat mass index	4.8 (0.4, 9.3)	0.03	5.0 (−0.2, 10.4)	5.1 (−0.6, 11.3)	0.95
Waist circumference	0.4 (−0.8, 1.5)	0.51	−0.1 (−1.4, 1.3)	1.3 (−0.2, 2.9)	0.12
HbA1c	1.9 (1.4, 2.4)	<0.0001	2.2 (1.6, 2.8)	1.7 (1.0, 2.4)	0.19
Glucose	−0.2 (−0.8, 0.4)	0.52	0.1 (−0.6, 0.9)	−0.4 (−1.2, 0.5)	0.33
Insulin	22.9 (16.8, 29.4)	<0.0001	20.0 (13.0, 27.4)	27.6 (19.3, 36.6)	0.09
Insulin resistance	22.4 (16.3, 28.7)	<0.0001	19.6 (12.6, 27.0)	27.0 (18.8, 35.8)	0.09
Triglyceride	−10.6 (−13.3, −7.7)	<0.0001	−13.3 (−16.4, −10.1)	−6.5 (−10.2, −2.5)	<0.001
HDL cholesterol	1.9 (0.2, 3.6)	0.03	1.9 (−0.1, 3.9)	1.2 (−1.0, 3.4)	0.58
C-reactive protein	21.0 (8.5, 35.0)	<0.001	19.6 (5.2, 36.0)	34.8 (16.6, 55.8)	0.14

“Ethnic differences” refer to differences from white Europeans and are adjusted for age quartiles, gender observer (physical measurements), month, and a random effect for school. Missing values: Fat mass index (*n* = 62), insulin and insulin resistance (*n* = 121), and C-reactive protein (*n* = 188).

*p* value B = *p* (no difference between black African-Caribbean subcategories).

### Risk Factors in South Asians and Other Asians

Compared with white Europeans, South Asian children were similar in height, but lighter. They had a lower mean ponderal index and waist circumference, but their mean sum of skinfolds, fat mass index, HbA1c, fasting glucose, fasting insulin, HOMA insulin resistance, C-reactive protein and triglyceride were higher, while HDL-cholesterol levels were lower (Tables 2 and 3, Figure 1). The differences in insulin, triglyceride, HDL-cholesterol, and fat mass index differed between South Asian subcategories, being larger in Bangladeshi children than among Pakistanis and Indians, though they were present among all three ethnic subcategories (Table 3). Glucose levels followed a similar pattern, though heterogeneity between ethnic subcategories was not statistically significant (Table 3). However, while HbA1c levels were higher among all South Asian subcategories, they were least raised among Bangladeshis (Table 3). Other Asians showed similar but less marked differences (Table 3). Compared with white Europeans, they had a higher sum of skinfolds and fat mass index, while other adiposity markers were similar. Levels of HbA1c, fasting insulin, HOMA insulin resistance, triglyceride, and C-reactive protein were higher (though less so than among South Asians) and HDL-cholesterol non-significantly lower. All differences observed were similar among boys and girls (unpublished data).

### Risk Factors in Black African-Caribbeans

Compared with white Europeans, black African-Caribbean children were on average markedly taller and heavier. However, their mean ponderal index, waist circumference, sum of skinfolds, and fat mass index were similar, while waist-hip ratio was slightly lower (Tables 2 and 4, Figure 1). In contrast, fasting insulin, HOMA insulin resistance, and HbA1c levels (but not fasting glucose) were higher in black African-Caribbeans (though not as high as in South Asians, particularly for insulin and insulin resistance); mean triglyceride was lower and HDL-cholesterol higher (in contrast with South Asians) (Table 4, Figure 1). Black Caribbean children tended to have higher levels of adiposity (particularly ponderal index) and higher triglyceride when compared with black Africans, while insulin, C-reactive protein, and other risk markers did not differ markedly between these groups (Table 4). All differences observed were similar among boys and girls (unpublished data).

### Effect of Adjustment for Adiposity

The effect of adjustment for adiposity (particularly for sum of skinfolds and fat mass index) on ethnic differences in insulin and glucose, blood lipids, and C-reactive protein was examined (Table 5). For South Asians, such adjustment had little effect on the size of HbA1c and glucose differences. Differences in fasting insulin, insulin resistance, triglyceride, HDL-cholesterol, and C-reactive protein were slightly reduced in size, but all remained strongly statistically significant. The pattern for other Asians was very similar, except that in this considerably smaller group adjustment for adiposity reduced the more modest initial differences in HDL-cholesterol and C-reactive protein markedly. For black African-Caribbeans, differences in HbA1c, insulin, insulin resistance, triglyceride, and HDL-cholesterol were not materially affected by adjustment for adiposity. The inclusion of other adiposity measures (particularly ponderal index and waist circumference) in adjustment reduced, rather than increased, the impact of adjustment for adiposity. The addition of C-reactive protein to adiposity adjustments (sum of skinfolds and fat mass index) for insulin, glucose, and blood lipids made no material difference to the results observed.

**Table 5:** Ethnic differences in blood markers (ethnic minority groups minus white Europeans): Effect of additional adjustment for adiposity (fat mass index and sum of skinfolds).

		South Asian (*n* = 1,290)		Other Asian (*n* = 291)		Black African-Caribbean (*n* = 1,205)	
Marker	Adjusted for Adiposity?	% Difference (95% CI)	*p* (Diff)	% Difference (95% CI)	*p* (Diff)	% Difference (95% CI)	*p* (Diff)
HbA1c	No	2.1 (1.6, 2.6)	<0.0001	0.9 (0.1, 1.7)	0.02	1.8 (1.3, 2.4)	<0.0001
	Yes	2.0 (1.5, 2.6)	<0.0001	0.8 (0.0, 1.6)	0.04	1.8 (1.3, 2.3)	<0.0001
Glucose	No	0.8 (0.1, 1.4)	0.02	0.8 (−0.2, 1.8)	0.11	−0.2 (−0.9, 0.4)	0.46
	Yes	0.7 (0.1, 1.4)	0.03	0.8 (−0.2, 1.8)	0.13	−0.2 (−0.8, 0.4)	0.56
Insulin	No	30.4 (23.7, 37.4)	<0.0001	24.1 (14.5, 34.6)	<0.0001	22.8 (16.6, 29.3)	<0.0001
	Yes	26.7 (21.0, 32.6)	<0.0001	19.6 (11.4, 28.4)	<0.0001	22.6 (17.2, 28.2)	<0.0001
Insulin resistance	No	29.9 (23.4, 36.8)	<0.0001	23.3 (13.7, 33.6)	<0.0001	22.2 (16.1, 28.6)	<0.0001
	Yes	26.2 (20.6, 32.1)	<0.0001	18.8 (10.7, 27.5)	<0.0001	22.0 (16.7, 27.6)	<0.0001
Triglyceride	No	13.3 (9.7, 16.9)	<0.0001	10.4 (5.1, 16.0)	<0.0001	−10.5 (−13.2, −7.7)	<0.0001
	Yes	11.9 (8.6, 15.4)	<0.0001	8.5 (3.6, 13.7)	<0.001	−10.7 (−13.3, −8.0)	<0.0001
HDL cholesterol	No	−3.0 (−4.6, −1.4)	<0.001	−1.8 (−4.3, 0.8)	0.17	1.9 (0.2, 3.6)	0.03
	Yes	−2.4 (−4.0, −0.9)	0.003	−0.9 (−3.4, 1.6)	0.47	2.0 (0.4, 3.7)	0.01
C-reactive protein	No	43.1 (28.3, 59.6)	<0.0001	18.0 (−0.9, 40.4)	0.06	19.6 (7.2, 33.4)	0.001
	Yes	32.2 (20.6, 44.9)	<0.0001	6.1 (−8.3, 22.7)	0.43	17.7 (7.4, 29.0)	<0.001

All ethnic differences are adjusted for age quartiles, gender, month, and a random effect of school. Missing values: insulin and insulin resistance (*n* = 115) and C-reactive protein (*n* = 182).

### Supplementary Analyses

The ethnic differences in HbA1c observed were not affected by the exclusion of children with abnormal haemoglobin variants or with increased amounts of normal variant fetal haemoglobin (HbF). The characteristics of participating children with complete fasting measurements (age, gender, social class, height, adiposity) were similar to those of children without such measurements. Additional adjustment for parental social class (related to height but not consistently to the other risk markers studied) had no appreciable effect on the ethnic differences observed (Tables S1, S2). Analyses which made adjustment for pubertal status or excluded children who showed evidence of pubertal development had no material effect on the ethnic differences observed. The ethnic differences in C-reactive protein levels were unaffected by the exclusion of children reporting a current health problem.

## Discussion

There is growing evidence that the higher prevalences of type 2 diabetes in ethnic minority groups (particularly South Asians) are apparent in young people as well as in middle age, both in the UK [14] and in the US [22],[23]. The present study extends these observations and preliminary findings in small numbers of South Asian (predominantly Pakistani) children and adolescents [15],[16],[24],[25] by demonstrating (for the first time in South Asian diaspora populations) that ethnic group differences in type 2 diabetes precursors are present in apparently healthy children at the end of the first decade. South Asian children have higher levels of HbA1c, insulin, triglyceride, and C-reactive protein and lower levels of HDL-cholesterol; the differences are particularly marked among children of Bangladeshi origin. The study also provides novel information on the risk profiles of UK black African-Caribbean children, who have higher levels of HbA1c, insulin, C-reactive protein, and (paradoxically) HDL-cholesterol, with lower levels of triglyceride. These patterns (particularly for insulin resistance) are consistent with differences between African-American and white European origin children in the US, both in the Bogalusa Heart Study [26] and in the nationwide NHANES Study [27].

The emerging differences in hyperglycaemia and insulin resistance correspond closely with established UK adult patterns of type 2 diabetes risk, markedly increased among South Asians and moderately increased among black African-Caribbeans; information on other Asian adults in the UK is very limited [2],[3],[5],[10]. The particularly marked insulin resistance observed among Bangladeshi children is consistent with the very high rates of type 2 diabetes among Bangladeshi adults [3]–[5]. Patterns of triglyceride and HDL-cholesterol (high-low in South Asians, low-high in black African-Caribbeans) are consistent with patterns observed in the respective UK adult populations [3]–[5] and (in the case of black African-Caribbeans) with patterns in African Americans in the US [28]; the different triglyceride and HDL-cholesterol patterns are clearly distinct from patterns of hyperglycaemia and insulin resistance, as previously noted [29]. The increased levels of C-reactive protein observed in both South Asians and black African-Caribbeans (associated with insulin resistance and the metabolic syndrome in both adults and children [7],[30]) are more consistent with the hyperglycaemia and insulin resistance observed in these children, and with the high levels of C-reactive protein reported in UK South Asian adults [3],[6],[8]. However, they are less consistent with levels in UK black African-Caribbean adults, which tend to be lower than in white Europeans [11].

The present study was designed to be sufficiently large to detect even modest (0.2 SD) differences in risk markers between major ethnic groups, and to include balanced representation of South Asian children of Indian, Pakistani, and Bangladeshi origin, and of black African-Caribbean children of African and Caribbean origin, sampled randomly from the eligible populations. The study included children from three UK cities which together account for more than two-thirds of all South Asians and black African-Caribbeans living in the UK. Inclusion of children of both white European and ethnic minority origin within all schools ensured that ethnic comparisons were carried out on a within-school basis. Classification of ethnicity was based primarily on self-reported parental ethnicity, which agreed closely with ethnicity defined using other approaches, particularly parental and grand-parental place of origin. Although the overall response rates (particularly for blood sampling) were only moderate, they were comparable with or higher than those in many previous studies [3],[5],[31]; the characteristics of respondents who provided complete data were not appreciably different from those respondents who did not. Response rates were, however, similar in all ethnic groups except black African-Caribbeans, and are not likely to have accounted for the risk marker patterns observed. It was particularly striking that the most marked risk factor differences occurred in South Asians and white Europeans, groups with very similar response rates. Ethnic differences in characteristics such as height between the ethnic groups (black African-Caribbeans markedly taller, South Asians shorter) are entirely consistent with those reported in earlier national surveys [3],[4],[32], supporting the representativeness of the population studied.

The ethnic differences in HbA1c, insulin, triglyceride, and C-reactive protein and lower levels of HDL-cholesterol observed in the present study did not appear to be accounted for by ethnic differences in adiposity, or by the influence of socioeconomic status. Most previous UK studies examining adiposity patterns in children from different ethnic groups have measured weight-for-height indices (particularly body mass index) [3],[4],[33], which appears to be unreliable for ethnic group comparisons [34]. In the present study, a wider range of adiposity markers suitable for school-based survey use were included, among which skinfold thickness measurements and bioelectrical impedance are particularly valuable for adiposity assessment [35]. Using these measurements, it appeared that South Asian children had higher levels of overall adiposity, a finding consistent with the results of a previous study using DXA measurements in slightly older children [36]. However, taking account of ethnic differences in adiposity defined by these measurements had little or no effect on the ethnic differences in insulin and other metabolic markers; additional adjustment for C-reactive protein (suggested to be a marker of adiposity complementary to anthropometric measurements [37]) had no additional effect. While further studies using gold standard adiposity markers are needed, the results suggest that the ethnic differences in diabetes precursors do not solely reflect ethnic differences in adiposity, consistent with earlier reports both in South Asians [15],[16] and in African Americans [38]. Earlier investigators have emphasized the potential importance of socioeconomic status in explaining ethnic differences in chronic disease risk [39]. In the present study population, socio-economic status, though positively related to height, was not consistently associated with the diabetes precursors measured. The absence of consistent associations between socioeconomic status and diabetes precursors in childhood is entirely consistent with reports from earlier UK studies [40],[41] and suggests that socioeconomic factors are not responsible for these emerging ethnic differences.

The ethnic differences in childhood insulin, triglyceride, HbA1c, and C-reactive protein levels observed here, though smaller (both relatively and absolutely) than those reported in adults [2],[3],[42], are appreciable in size (respectively, 30%, 13%, 2%, and 43% differences between South Asians and white Europeans). The results imply that ethnic differences in type 2 diabetes in the UK, originally described in first generation immigrants, are persisting in UK-born South Asian and black African-Caribbean immigrants (more than 80% of study participants in all the main ethnic groups in the present study were UK born). They also suggest that at least some of the aetiological factors responsible for ethnic differences in insulin resistance and type 2 diabetes are operating well before adult life, potentially offering opportunities for early prevention. The observations could be relevant not only in the UK but in other settings in which ethnic differences in type 2 diabetes emerge at an early age. In the United States, for example, high risks of type 2 diabetes are apparent in African Americans and Asian Americans in the second decade of life [13],[22],[23]. Relevant exposures could be operating in childhood, in infancy, or in fetal life [12], though the contributions of specific exposures may well differ between specific ethnic groups. Lower levels of physical activity in childhood could be important, particularly among South Asians [43]; childhood diet could also be relevant. Ethnic differences in early postnatal growth could play an important role [44]. Fetal undernutrition and low birth weight are related to type 2 diabetes risk [45] and are highly prevalent among UK South Asians (particularly Bangladeshis) [46]. Fetal undernutrition could lead to ethnic differences in body composition and insulin resistance very early in life [47], though this has not yet been examined in UK South Asians. Ethnic differences in maternal factors influencing type 2 diabetes risk in offspring (particularly maternal obesity and gestational hyperglycaemia) [48],[49] could also play an important role.

The implications of the results for prevention require careful consideration. The new epidemic of early onset type 2 diabetes occurring in many Western societies affects all sections of the population [50], and key preventive measures in childhood (particularly directed to increasing physical activity levels, improving dietary nutrient content, and preventing overweight and obesity) are likely to be widely desirable [50]. However, there is a particularly urgent need for preventive measures in high-risk ethnic groups [1],[14], in which the benefits of prevention are potentially greater [51]. Since such prevention includes educational elements, it is likely to address culturally sensitive issues. Such interventions may therefore need to be specifically tailored to the needs of particular ethnic groups [52].

## Supporting Information

Table S1 Ethnic differences in physical measurements and blood markers (South Asian and Asian other – white Europeans), including adjustment for socio-economic status.
(0.06 MB PDF)

Table S2 Ethnic differences in physical measurements and blood markers (Black African-Caribbean - white Europeans), including adjustment for socio-economic status.
(0.06 MB PDF)

## Abbreviations

CHASE: Child Heart and Health Study in England
DXA: dual energy X-ray absorptiometry
HOMA: homeostasis model assessment
