# Supplementary material for: Early Emergence of Ethnic Differences in Type 2 Diabetes Precursors in the UK: The Child Heart and Health Study in England (CHASE Study)
Source: PLoS Med. 2010 Apr 20;7(4):e1000263. doi: 10.1371/journal.pmed.1000263 (PMC2857652; doi:10.1371/journal.pmed.1000263)
Supplement: Table S1 — Ethnic differences in physical measurements and blood markers (South Asian and Asian other – white Europeans), including adjustment for socio-economic status. (0.06 MB PDF) [file pmed.1000263.s001.pdf]

Table S1: Ethnic differences in physical measurements and blood markers (South Asian and Asian other – white Europeans) including adjustment for socio-economic status

				South Asian subcategories									
	All South Asian (n=1093)			Indian (n=372)		Pakistani (n=392)		Bangladeshi (n=237)			All Other Asian (n=228)		
	% difference	(95% CI)	p (diff)	% difference	(95% CI)	% difference	(95% CI)	% difference	(95% CI)	p-value A	% difference	(95% CI)	p (diff)
Height	-0.3	(-0.7, 0.1)	0.18	-0.1	(-0.7, 0.4)	0.1	(-0.5, 0.7)	-0.9	(-1.6, -0.2)	0.05	-0.7	(-1.4, 0.0)	0.05
Weight	-2.8	(-4.7, -0.8)	0.01	-3.5	(-6.2, -0.7)	-2.3	(-5.0, 0.5)	-1.8	(-5.0, 1.6)	0.66	-1.0	(-4.3, 2.4)	0.56
Ponderal Index	-1.9	(-3.3, -0.5)	0.01	-3.1	(-5.0, -1.1)	-2.5	(-4.5, -0.6)	0.9	(-1.5, 3.3)	0.02	1.1	(-1.3, 3.6)	0.36
Sum of skinfolds	5.1	(0.7, 9.7)	0.02	4.2	(-1.8, 10.6)	6.2	(0.2, 12.6)	6.5	(-0.8, 14.3)	0.85	2.4	(-4.6, 9.9)	0.51
Fat mass index	7.8	(2.8, 13.0)	0.002	3.5	(-3.1, 10.6)	6.7	(0.0, 13.8)	14.9	(6.3, 24.2)	0.08	7.3	(-0.7, 15.9)	0.07
Waist circumference	-1.4	(-2.7, -0.2)	0.02	-2.2	(-3.8, -0.5)	-1.0	(-2.7, 0.6)	-0.9	(-2.9, 1.2)	0.45	-0.4	(-2.4, 1.7)	0.71
HbA1c	2.3	(1.7, 2.8)	<0.0001	2.8	(2.0, 3.6)	2.6	(1.8, 3.4)	1.1	(0.1, 2.0)	0.004	0.9	(0.0, 1.9)	0.04
Glucose	0.8	(0.1, 1.5)	0.02	0.6	(-0.3, 1.6)	1.0	(0.1, 2.0)	1.6	(0.4, 2.7)	0.37	0.5	(-0.6, 1.6)	0.36
Insulin	30.4	(23.2, 37.9)	<0.0001	28.9	(19.2, 39.4)	22.0	(13.0, 31.8)	49.8	(36.6, 64.3)	<0.001	21.0	(10.4, 32.5)	<0.0001
Insulin resistance	30.0	(22.9, 37.5)	<0.0001	28.0	(18.4, 38.3)	22.1	(13.2, 31.8)	49.2	(36.2, 63.5)	<0.001	21.4	(10.9, 32.8)	<0.0001
Triglyceride	12.7	(8.9, 16.6)	<0.0001	10.8	(5.7, 16.1)	12.0	(6.9, 17.4)	18.9	(12.4, 25.7)	0.09	8.3	(2.4, 14.4)	0.005
HDL cholesterol	-2.9	(-4.6, -1.1)	0.002	-1.6	(-4.0, 0.8)	-1.1	(-3.5, 1.4)	-7.3	(-10.0, -4.5)	<0.001	-0.7	(-3.6, 2.2)	0.62
C reactive protein	45.2	(29.1, 63.3)	<0.0001	43.3	(21.7, 68.6)	52.7	(30.1, 79.2)	36.6	(12.7, 65.5)	0.56	21.6	(0.0, 47.8)	0.05

‘Ethnic differences’ refer to differences from white Europeans and are adjusted (with the exception of age) for age quartiles, gender observer (physical measurements), month, socio-economic status (SOC2000), and a random effect for school
p-value A = p (no difference between South Asian sub groups)
Missing values: Fat mass index (n=52), Insulin and Insulin resistance (n=101), C reactive protein (n=160)
