# Supplementary material for: Early Emergence of Ethnic Differences in Type 2 Diabetes Precursors in the UK: The Child Heart and Health Study in England (CHASE Study)
Source: PLoS Med. 2010 Apr 20;7(4):e1000263. doi: 10.1371/journal.pmed.1000263 (PMC2857652; doi:10.1371/journal.pmed.1000263)
Supplement: Table S2 — Ethnic differences in physical measurements and blood markers (Black African-Caribbean - white Europeans), including adjustment for socio-economic status. (0.06 MB PDF) [file pmed.1000263.s002.pdf]

Table S2: Ethnic differences in physical measurements and blood markers (Black African-Caribbean – white Europeans) including adjustment for socio-economic status

	All Black African-Caribbean (n=960)			Black African-Caribbean subcategories<br>Black African (n=489)		Black Caribbean (n=405)			All Other (n=714)		
	% difference	(95% CI)	p (diff)	% difference	(95% CI)	% difference	(95% CI)	p-value B	% difference	(95% CI)	p (diff)
Weight	2.9	(2.5, 3.4)	<0.0001	2.9	(2.3, 3.4)	3.0	(2.4, 3.5)	0.84	0.8	(0.3, 1.3)	<0.001
Weight	11.0	(8.7, 13.4)	<0.0001	10.0	(7.2, 12.8)	12.7	(9.7, 15.8)	0.12	3.9	(1.7, 6.3)	<0.001
Ponderal Index	1.8	(0.3, 3.4)	0.02	0.9	(-0.9, 2.8)	3.3	(1.3, 5.3)	0.04	1.5	(-0.1, 3.1)	0.07
Sum of skinfolds	-1.3	(-5.5, 3.2)	0.57	-1.1	(-6.3, 4.3)	0.4	(-5.1, 6.3)	0.62	1.9	(-2.8, 6.7)	0.44
Fat mass index	7.2	(2.1, 12.4)	0.005	7.0	(1.0, 13.5)	8.4	(1.8, 15.3)	0.70	6.3	(1.0, 11.8)	0.02
Waist circumference	0.8	(-0.4, 2.1)	0.20	0.3	(-1.3, 1.9)	1.9	(0.2, 3.5)	0.11	0.6	(-0.7, 2.0)	0.36
HbA1c	2.1	(1.5, 2.6)	<0.0001	2.3	(1.6, 3.0)	1.9	(1.2, 2.6)	0.28	0.8	(0.2, 1.4)	0.01
Glucose	-0.4	(-1.1, 0.3)	0.24	-0.2	(-1.0, 0.6)	-0.3	(-1.2, 0.6)	0.86	-0.3	(-1.0, 0.4)	0.38
Insulin	25.8	(18.9, 33.2)	<0.0001	22.6	(14.4, 31.3)	30.7	(21.5, 40.7)	0.11	16.8	(10.1, 24.0)	<0.0001
Insulin resistance	25.4	(18.6, 32.7)	<0.0001	22.5	(14.4, 31.2)	30.2	(21.1, 39.9)	0.13	16.5	(9.9, 23.6)	<0.0001
Triglyceride	-9.7	(-12.8, -6.6)	<0.0001	-12.9	(-16.5, -9.2)	-5.7	(-9.8, -1.4)	0.002	0.9	(-2.7, 4.6)	0.64
HDL cholesterol	1.8	(0.0, 3.7)	0.05	2.1	(-0.1, 4.4)	1.1	(-1.3, 3.5)	0.44	0.1	(-1.8, 2.0)	0.94
C reactive protein	29.2	(14.5, 45.6)	<0.0001	26.9	(9.6, 46.9)	41.4	(21.0, 65.2)	0.22	20.8	(6.3, 37.3)	0.004

'Ethnic differences' refer to differences from white Europeans and are adjusted (with the exception of age) for age quartiles, gender observer (physical measurements), month, socio-economic status (SOC2000), and a random effect for school
p-value B = p (no difference between Black sub groups)
Missing values: Fat mass index (n=52), Insulin and Insulin resistance (n=101), C reactive protein (n=160)
